# Tailored Magnetic Multicore Nanoparticles for Use as Blood Pool MPI Tracers

**DOI:** 10.3390/nano11061532

**Published:** 2021-06-10

**Authors:** Harald Kratz, Azadeh Mohtashamdolatshahi, Dietmar Eberbeck, Olaf Kosch, Frank Wiekhorst, Matthias Taupitz, Bernd Hamm, Nicola Stolzenburg, Jörg Schnorr

**Affiliations:** 1 Department of Radiology, Charité-Universitätsmedizin Berlin, Corporate Member of Freie Universität Berlin and Humboldt-Universität zu Berlin, D-10117 Berlin, Germany; Azadeh.Mohtashamdolatshahi@charite.de (A.M.); Matthias.Taupitz@charite.de (M.T.); Bernd.Hamm@charite.de (B.H.); Nicola.Stolzenburg@charite.de (N.S.); Joerg.Schnorr@charite.de (J.S.); 2 Physikalisch-Technische Bundesanstalt, D-10587 Berlin, Germany; Dietmar.Eberbeck@ptb.de (D.E.); Olaf.Kosch@ptb.de (O.K.); Frank.Wiekhorst@ptb.de (F.W.)

**Keywords:** magnetic particle imaging (MPI), magnetic particle spectroscopy (MPS), magnetic nanoparticles (MNP), magnetic multicore particles (MCP), coprecipitation, polyethylene glycol (PEG), blood half-life

## Abstract

For the preclinical development of magnetic particle imaging (MPI) in general, and the exploration of possible new clinical applications of MPI in particular, tailored MPI tracers with surface properties optimized for the intended use are needed. Here we present the synthesis of magnetic multicore particles (MCPs) modified with polyethylene glycol (PEG) for use as blood pool MPI tracers. To achieve the stealth effect the carboxylic groups of the parent MCP were activated and coupled with pegylated amines (mPEG-amines) with different PEG-chain lengths from 2 to 20 kDa. The resulting MCP-PEG variants with PEG-chain lengths of 10 kDa (MCP-PEG10K after one pegylation step and MCP-PEG10K2 after a second pegylation step) formed stable dispersions and showed strong evidence of a successful reaction of MCP and MCP-PEG10K with mPEG-amine with 10 kDa, while maintaining their magnetic properties. In rats, the mean blood half-lives, surprisingly, were 2 and 62 min, respectively, and therefore, for MCP-PEG10K2, dramatically extended compared to the parent MCP, presumably due to the higher PEG density on the particle surface, which may lead to a lower phagocytosis rate. Because of their significantly extended blood half-life, MCP-PEG10K2 are very promising as blood pool tracers for future in vivo cardiovascular MPI.

## 1. Introduction

Magnetic nanoparticle (MNP) based on iron oxides have been the subject of medical research and development for decades. They have many applications including magnetically induced hyperthermia for cancer treatment [1,2], iron replacement therapy [3], bioassays [4,5] and as contrast agents for medical and molecular imaging by magnetic resonance imaging (MRI) [6,7] or magnetic particle imaging (MPI) [8]. MNP used as contrast agents typically consist of an iron oxide core with an appropriate coating for stabilization. The iron oxide core itself predominantly consists of magnetite, maghemite or a magnetite/maghemite mixed phase. In addition, their magnetic characteristics for use as contrast agents for MRI or as MPI tracers must meet specific criteria. MPI was introduced by Weizenecker and Gleich in 2005 as a new radiationless imaging modality which provides 4D imaging with high temporal resolution (46 frames per second) and a spatial resolution on the order of 1 mm, depending on the type of MPI scanner and MPI tracer used [9]. Since only the dynamic magnetic response of the MNP exposed to an oscillating excitation field is inductively detected, the surrounding tissue does not contribute any signal to the MPI image. Therefore, certain medical applications crucially rely on the combination of MPI with other imaging modalities like MRI to obtain related anatomical information [10]. MPI can quantitatively determine tracer distribution and distinguish between different binding states of the MNP, for example, if the particles are unbound in dispersion, bound to a surface or are taken up by cells [11,12,13]. Promising imaging applications for preclinical imaging are stem cell tracking [14,15] and immune cell tracking [16,17] as well as imaging of the cardiovascular system [17,18,19,20] and local perfusion [21,22]. To address these applications and to find possible new clinical applications, it is pivotal to develop tailored MPI tracers with specific properties. Magnetic multicore particles (MCPs) were developed for MPI by a modified cost-effective coprecipitation method. Monodispersity, which is crucial for a good MPI performance, is achieved by fractional magnetic separation and the very good magnetic properties, by an annealing process [9,23,24]. In MCPs, small single crystallite cores are combined to form larger nanoparticles, which have already shown very good MPI tracer performance along with excellent in vivo behavior in rats [25,26,27,28]. MCPs are coated with carboxymethyl dextran (CMD) and therefore negatively charged at physiological pH. The negative ζ-potential of about −33.5 mV leads to an increased cellular uptake of these particles compared to MNP with an uncharged surface [25,29]. The high cellular uptake enables the use of MCPs for potential single cell tracking using MPI or MRI [30]. On the other hand, due to enhanced uptake in the liver and spleen, the in vivo blood half-life after intravenous (i.v.) injection is limited, and therefore, their use as MPI tracers for imaging of the cardiovascular system is also limited [17,18,19]. The negative charge of MCPs is attributable to carboxyl groups located on their surface, which can be used for coupling reactions and therefore for modulating MNP surface characteristics [25]. A very common method to achieve an extended in vivo blood half-life is to coat the MNP surface with polyethylene glycol (PEG) [31]. This technique is well established for the modification of nanoparticles and also proteins, among other things [32,33,34,35]. PEG has a very high water-binding capacity and hence strongly reduces interaction of the particle surface with proteins, which in turn leads to a reduced recognition of the respective pegylated MNP by the immune system [31]. Our hypothesis was therefore that it should be possible to anchor PEG derivatives on the surface of MCPs to achieve a prolonged in vivo blood half-life. In the following we describe the development of these pegylated MCPs, their physicochemical characterization and first in vivo blood half-life studies using MRI.

## 2. Materials and Methods

### 2.1. Chemicals

Unless otherwise noted, all Chemicals were purchased from Sigma-Aldrich (Steinheim, Germany) and were used without any further purification. Methoxy polyethylene glycol amine (mPEG-amine) with molecular weights of 2 kD, 5 kD, 10 kD and 20 kD (mPEG-amine 2K–20K) were purchased from Laysan Bio. Inc. (Arab, AL, USA). Deionized water was produced using a Milli-Q A10 system (Millipore, Billerica, MA, USA) and was used to prepare all solutions and dispersions.

### 2.2. MPI Tracers

Here, the syntheses of 3 variants of pegylated MCPs using 5 kD and 10 kD mPEG-amine, leading to MCP-PEG5K, MCP-PEG10K and MCP-PEG10K2 are described. The syntheses of pegylated MCPs using 2 kD and 20 kD mPEG-amine, leading to MCP-PEG2K and MCP-PEG20K, are described in supplement Protocols S1 and S2.

#### 2.2.1. MCPs

MCPs were synthesized in our laboratory according to a previously published synthesis protocol and are coated with carboxymethyl dextran (CMD) [25,26]. After synthesis, the MCPs were concentrated to approx. 300 mmol Fe/L by centrifugation with 3112× *g* using Amicon Ultra-15 Centrifugal Filter Units (PLHK Ultracel-PL Membrane, 100 kDa). MNP dispersions were diluted with Milli-Q water to prepare the respective final concentrations and stored in a fridge at 4 °C.

#### 2.2.2. Synthesis of MCP-PEG5K and MCP-PEG10K

An amount of 1.80 mL MCP dispersion (108.4 mM Fe/L) was subsequently mixed with a solution of 0.180 g (829 µmol) *N*-hydroxysulfosuccinimide sodium salt (sulfo-NHS) in 0.45 mL water and a solution of 0.54 g (2.817 mmol) *N*-(3-dimethylaminopropyl)-*N*′-ethylcarbodiimide hydrochloride (EDC) in 1.35 mL water. Thereafter, the mixture was agitated for 30 min at room temperature (RT) in a Roto-Therm Plus rotary blender (Benchmark, Sayreville, NJ, USA). The particle dispersion was then centrifuged at 780× *g* for 4 s, and 3.6 mL of the supernatant was removed and replaced by 3.6 mL of water and the mixture was vortexed. The particle dispersion was then centrifuged a second time at 780× *g* for 4 s and 3.6 mL of the supernatant was removed, 3.6 mL of water was added, and the mixture was vortexed again. Following these steps, the dispersion was added to a solution of 0.9 g (180 µmol) mPEG-amine5K (5 kD) in 3.6 mL of water for MCP-PEG5K or 1.8 g (180 µmol) mPEG-amine10K (10 kD) in 3.6 mL of water for MCP-PEG10K. After thorough mixing, the resulting batch was sonicated for 99 min using a Sonorex Digiplus DL 512 H Sonicator (Bandelin, Berlin, Germany) at 100% intensity (the temperature rose from RT to 48 °C) and then mixed at RT with a Roto-Therm Plus rotary blender at RT overnight. Water was then added to reach a total volume of about 30 mL, and the dispersion was concentrated to a volume of about 1 mL (2 × 500 µL) using two centrifuge filters (100 kD Ultracel Amicon Ulta 15,) at 3939× *g*. Then 3 mL of water were added in each case, and the dispersion was again concentrated to about 500 µL of volume at 3939× *g*. The last step, addition of water and subsequent centrifugation, was repeated three more times, and the volume of the resulting dispersion was collected and increased to a total volume of 1.1 mL by adding water. Iron content for MCP-PEG5K: 149 mM Fe/L (yield: 84% related to Fe); Iron content for MCP-PEG10K: 146 mM Fe/L (yield: 82% related to Fe).

#### 2.2.3. Synthesis of MCP-PEG10K2 by Renewed Conversion of MCP-PEG10K with mPEG-amine10K

An amount of 0.60 mL MCP-PEG10K dispersion (116.6 mM Fe/L) was subsequently mixed with a solution of 0.060 g (276 µmol) sulfo-NHS in 0.15 mL water and a solution of 0.18 g (0.939 mmol) EDC in 0.45 mL water. Thereafter, the mixture was agitated for 30 min at RT in a rotary blender. The particle dispersion was then concentrated using two centrifuge filters (100kD, RC, Ultracel, Amicon Ultra 0.5) at 9900× *g* to a volume of 180 µL (2 × 90 µL). The resulting dispersion was diluted with water to a total volume of 1 mL and then added to a solution of 0.6 g (60 µmol) mPEG-amine10K (10 kD) in 1.2 mL of water. After thorough mixing, the resulting batch was sonicated for 99 min using a Sonorex Digital 10 P Sonicator (Bandelin, Berlin, Germany) at 100% of intensity (the temperature rose from RT to 50 °C) and then mixed at RT with a Roto-Therm Plus rotary blender (Benchmark, Sayreville, NJ, USA) at RT overnight. Water was then added to reach a total volume of about 20 mL, and the dispersion was concentrated to a volume of about 0.8 mL (2 × 400 µL) using two centrifuge filters (100 kD, Ultracel Amicon Ulta 4,) at 3372× *g*. Then 3 mL of water were added in each case, and the dispersion was again concentrated to about 300 µL of volume at 3372× *g*. The last step, addition of water and subsequent centrifugation, was repeated three more times, and the volume of the resulting dispersion was collected and increased to a total volume of 0.4 mL by adding water. Iron content for MCP-PEG10K2: 163 mM Fe/L (yield: 93% related to Fe).

### 2.3. Nanoparticle Characterization

The iron content of the particle dispersions was colorimetrically determined using the phenanthroline method [36]. The hydrodynamic diameter distribution, weighted by volume (d_V_), the intensity-weighted mean hydrodynamic size (Z-Average), the polydispersity index (PDI), and the ζ-potential of the MNP were determined by dynamic light scattering (DLS) on a Zetasizer Nano ZS particle analyzer (Malvern Instruments, Worcestershire, UK). For DLS, PDI and Z-Average measurement, MNP dispersions were diluted with Milli-Q water to a final concentration of 1 mmol Fe/L. For ζ-potential measurement, MNP dispersions were diluted with 10 mM NaCl to a final concentration of 1 mmol Fe/L and adjusted to a pH of about 7.20 with NaOH. Short- and long-term stability of the MNP dispersions were investigated by visual inspection and by DLS. Nanoparticle size, morphology and phase were analyzed by transmission electron microscopy (TEM) using a TECNAI G2 20 S-Twin and a TITAN 80–300 (FEI Company, Hillsboro, OR, USA). ^1^H-NMR T1- and T2-relaxation rates were measured with a Minispec MQ 40 Time-Domain Nuclear magnetic resonance (TD-NMR) spectrometer at 40 °C and 0.94 T (Bruker, Karlsruhe, Germany). Relaxivities (relaxation coefficients) r1 and r2 were determined by linear fitting of T1- and T2-relaxation rates as functions of iron concentrations. MNP were also analyzed by magnetic particle spectroscopy (MPS) to obtain information on their response to alternating magnetic fields. MPS measurements were performed on undiluted samples using a magnetic particle spectrometer (MPS-3, Bruker BioSpin, Germany) at 12 mT, 25 kHz and 37 °C for 10 s. For measurement the samples were filled in Life Technologies polymerase chain reaction (PCR) tubes with sample volumes of 30 μL. The amplitude of the magnetic moment was normalized to the iron content of each sample, resulting in the spectrum of the magnetization, *M_k_*, which is given in Am^2^/mol(Fe).

To obtain an adequate reference for the immobilized state of MCP-PEG10K2 in organs, MNP were immobilized in polyacrylamide gel (PAA). For this, 94 µL acrylamide solution (30% in water), 94 µL *N*-*N′*-methylenebisacrylamide solution (2% in water), 9.5 µL ammonium persulfate (1% in water), 24 µL water, 60 µL MCP-PEG10K2 dispersion (10.6 mmol (Fe)/L), and 18.5 µL *N,N,N′,N′*-tetramethyletylenediamine (1:30 diluted with water (*v*/*v*)) were mixed and subsequently vortexed. Finally, 50 µL of the resulting dispersion was filled in a measuring cuvette and polymerized at 60 °C in a water bath for approx. 3 min. For M(H) measurement, 75 µL sample volumes were filled in a polycarbonate capsule. The magnetic moment of each sample was measured using an MPMS (Magnetic Property Measurement System, Quantum Design, San Diego, CA, USA) consecutively increasing the applied magnetic field from 0 to 5 T. The background signal caused by empty capsules, diamagnetic susceptibility of the dispersion medium, and demineralized water, was subtracted from the signal obtained for the samples. The resulting data represent MNP magnetization and were normalized to the iron content of the sample, which allows quantitative evaluation of the data. Since the M(H) did not saturate even at highest measurement fields, the value of *M,* at *H* = 448 kA/m was taken as a substitute of the saturation magnetization (*M*_S_). This field value was found to be a good compromise between a small change of *M* with *H* and a low uncertainty. Note that the uncertainty increases with the field value because of potential errors during background subtraction. Because of possible small deviation of the sample from the center position in the MPMS, we give an uncertainty of the absolute values of 5%. FTIR measurements were performed using a Bruker ALPHA spectrometer (model A250/D, Bruker Optik GmbH, Leipzig, Germany) equipped with a diamond ATR sampling module (model A220/D-01, Bruker Optik GmbH, Leipzig, Germany). Measured data were processed using the OPUS 6.5 software package (Bruker Optik GmbH, Leipzig, Germany). Background and baseline correction as well as atmospheric compensation were applied to all spectra. Spectra were acquired in absorbance mode and converted to transmission mode from 23 coadded scans between 4000 and 375 cm^−1^.

### 2.4. In Vivo MRI Studies

#### 2.4.1. In Vivo MR Imaging for Determination of MNP Blood Half-Life and Organ Distribution

Rats were kept in Type IV Macrolon1 cages (Zoonlab, Castrop-Rauxel, Germany) on softwood granulate (Lignocel, J. Rettenmaier, Rosenberg, Germany) at a constant 12-h day/night cycle, a temperature of 21 ± 1 °C, and 50 ± 5% relative humidity according to recommendation 2007/526/EC of the European Commission. Rats received commercial standard pellet feed (ssniff, R-M-H, Soest, Germany) and tap water ad libitum. In vivo experiments in rats were conducted in accordance with the requirements and guidelines of EU directive 2010/63/EU and the German Animal Protection Act. The animal studies were approved by the State Office of Health and Social Affairs Berlin (LAGESO) and were carried out in accordance with institutional and federal animal care guidelines. Images were acquired on a clinical 1.5 Tesla (Magnetom Sonata; Siemens Healthcare Solutions, Erlangen, Germany) and a 3 Tesla MRI scanner (Magnetom Lumina, Siemens Healthcare Solutions, Erlangen, Germany) using the 18-channel extremity coil (note: the initially used MRI scanner was replaced during the conduct of the study). In vivo examinations were carried out on 5 healthy male Sprague Dawley rats (Charles River Laboratories, Sulzfeld, Germany), 12 weeks old, with an average body weight of 355 ± 15 g. Prior to MRI, rats were anesthetized with 5% isoflurane in an anesthetic induction chamber and maintained in 1–2% isoflurane during MRI acquisitions. The MNP dispersions were injected into a lateral tail vein at a final dose of 50 µmol Fe/kg of bodyweight as a bolus over 2 s. Imaging was performed in coronal orientation with a T1-weighted 3D fast low-angle shot (FLASH) sequence every 5 to 10 min after MNP administration over a 90-min period. Further imaging parameters were: repetition time (TR) = 6.57 ms (at 3T) and TR = 5.7 ms (at 1.5 T), echo time (TE) = 1.95 ms, flip angle (FA) = 25°, field of view (FOV) = 300 × 300 mm, matrix size = 784 × 896 (reconstructed pixel size = 0.3 × 0.3 mm) and slice thickness = 0.6 mm, 80 slices, bandwidth of 130 Hz/Px (at 3T) and 200 Hz/Px (at 1.5 T). Qualitative biodistribution of MNP in liver and spleen was assessed using T2 * weighted images acquired in transverse orientation with a spoiled gradient echo (GRE) sequence before and after MNP administration (24 h post). Further imaging parameters were: TR/TE = 309, 4.02 ms, FA = 25°, FOV = 200 × 200 mm, matrix size = 288 × 288 (pixel size = 0.7 × 0.7 mm) and slice thickness = 1.5 mm, 18 slices, bandwidth of 404 Hz/Px (at 3T). MNP blood half-life was assessed in three regions of interest (ROIs) defined over the inferior vena cava of each animal at each time point up to 90 min after injection. From the average value in the ROIs at each time point, half-life was calculated as first-order exponential decay kinetics using GraphPad Prism software version 7 (GraphPad Software, San Diego, CA, USA).

#### 2.4.2. Quantification of MNP Biodistribution by MPS

Ex vivo MPS measurements of removed organs were performed in a commercial magnetic particle spectrometer (Bruker, Germany) with sinusoidal magnetic signal excitation using an amplitude of 25 mT, a frequency of 25 kHz and a sample temperature of 37 °C. The nonlinear magnetization response of MNPs in organs was measured for 10 s by a pickup coil (sensitivity: 10^–12^ Am^2^). All MPS sample measurements were corrected by subtraction of the background signal of the empty sample holder from the MPS spectra. For MPS measurement the removed organs were cut into small pieces using a ceramic scalpel to avoid iron contamination. A PAA-embedded sample of MCP-PEG10K2 was used as a reference for iron quantification, which showed a close match of spectra (by the MPS shape parameter A5/A3) with the tissue samples. For quantification, the amplitude of the 3rd harmonic of the MPS spectra of measured samples was normalized to the amplitude of the reference sample (for MPS spectrum of this reference, please see supplement Figure S6).

## 3. Results and Discussion

### 3.1. Nanoparticle Synthesis and Characterization

**Figure 1:**
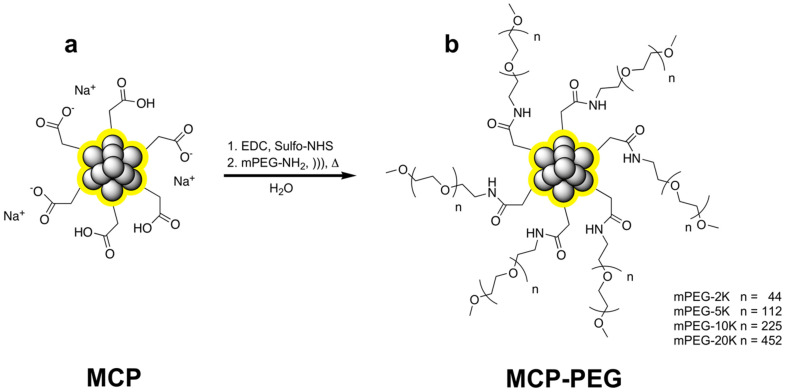
Activation of MCPs (**a**) with EDC and sulfo-NHS and subsequent conversion with mPEG-amines of different chain lengths to obtain the corresponding MCP-PEG variants (**b**).

In this work, we synthesized pegylated MCPs (MCP-PEG) by activation of the carboxyl groups of MCPs using an EDC/sulfo-NHS strategy and subsequent conversion of the intermediately formed sulfo-NHS active ester with mPEG-amines of different chain lengths (Figure 1).

Synthesis was performed with different mPEG-amine derivatives with molecular weights of 2, 5, 10 and 20 kD molecular weight. Depending on the molecular weight of the converted mPEG-amines and to prevent aggregation of the MNP during conversion the reactions were performed in an ultrasonic bath and, in some cases, at higher temperatures. Because of the sensitivity of PEG and CMD to high intensity ultrasound, a low-energy ultrasonic cleaning bath was used to prevent degradation of the coating polymers [37,38,39]. Dispersion stabilities of the MCP-PEG variants obtained by conversion are shown in Table 1.

**Figure 2:**
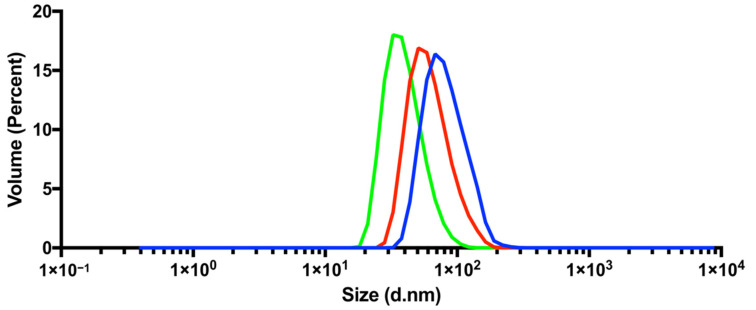
DLS results for MCPs (green), MCP-PEG10K (red) and MCP-PEG10K2 (blue). Graphs represent mean of 6 volume measurements. (For size distribution by intensity and DLS results of MCP, MCP-PEG5K, MCP-PEG10K, MCP-PEG10K2 and MCP-PEG20K please see supplement Figures S1–S3).

For MCP-PEG2K, we failed to achieve stable dispersions, which may be attributable to the relatively low stabilizing effect of the short PEG chain. In contrast, for MCP-PEG5K, we initially obtained stable dispersions, but then observed sedimentation within a period of about 6 h. Similar results were obtained after conversion of the MCPs with mPEG-amine20K. The dispersions were not stable, and sedimentation occurred after about 2 h. The reasons for the sedimentation of MCP-PEG5K and MCP-PEG20K are probably due to aggregation occurring during synthesis, because mPEG-amine5K does not stabilize sufficiently and mPEG-amine20K probably reacts too slowly due to the chain lengths. The only promising results were achieved using mPEG-amine10K, which led to stable MCP-PEG10K dispersions with a trend to form a concentration gradient after several days but without any changes in hydrodynamic diameter measured by DLS. Therefore, we decided to exclusively use MCP-PEG10K for further work. To investigate the possibility of physisorption of mPEG-amine10K to the MCP surface, instead of amide formation, the same reaction conditions were applied in control experiments, but sulfo-NHS and EDC were omitted in one case and EDC in the other. The results of the first experiment were unchanged MCPs and in the second the pronounced formation of aggregates was observed. The next step in this study was to convert MCP-PEG10K with mPEG-amine10K a second time to further optimize the MPI tracers blood half-life by increasing PEG density on the MCPs surfaces. The resulting MCP-PEG10K2 dispersions were stable without a tendency for gradient formation. DLS measurements in general showed a significant increase in hydrodynamic diameters of the mPEG-amine-converted MCPs compared with the parent MCPs. MCP-PEG20K displayed a very strong increase in hydrodynamic diameters and different from all other pegylated MNPs, a high proportion of aggregates could be detected. (For DLS results of MCP-PEG5K and MCP-PEG20K, please see supplement Figures S2 and S3.) The results of the DLS measurements of MCPs, MCP-PEG10K and MCP-PEG10K2 by volume are shown in Figure 2. Hydrodynamic diameters increased with each conversion, probably indicating higher PEG surface density in each case.

Z-Average values also increased with every conversion, and ζ-potentials decreased from MCP to the pegylated MCP because of the reduced surface charge after the reaction with mPEG-amine10K (Table 2). A further decrease in Zeta potential value would also be expected after the second pegylation, but with such small values close to zero, large deviations can occur due to the large pH value sensitivity.

**Figure 3:**
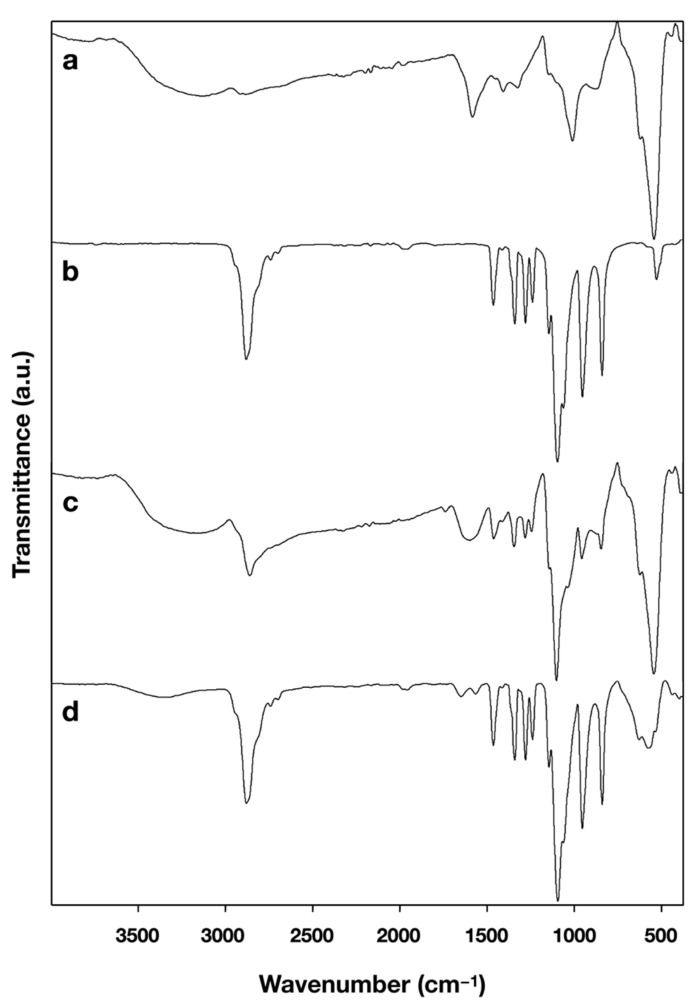
(**a**–**d**). FTIR spectra of (**a**) MCP, (**b**) mPEG-amine10K, (**c**) MCP-PEG10K and (**d**) MCP-PEG10K2. For a list of the characteristic FTIR absorption bands and their assignment please see supplement Tables S4–S7.

Given in Table 2 are relaxivities r1 and r2, measured by TD-^1^H NMR. Also shown are the mean hydrodynamic diameter by volume (dv), the intensity-weighted mean hydrodynamic size (Z-Average), the polydispersity index (PDI) and the zeta potential (ζ), all measured by DLS. Conversion of the activated MNP surface with the mPEG-amines could be examined by FTIR measurement for MCP-PEG10K and MCP-PEG10K2 (Figure 3). The mPEG-amine-specific bands are clearly detectable in MCP-PEG10K and even more markedly in MCP-PEG10K2, which might indicate a higher PEG density on the MNP surfaces after the second conversion. Furthermore, there are two bands at 1565 cm^−1^ and 1649 cm^−1^ in the FTIR spectrum of MCP-PEGK2 (Figure 3d), which can be attributed to amide bonds, which are formed during the pegylation reaction [40,41]. Please note that these bands (Amine I and II) are not very pronounced, because there is only one amino group per PEG chain with a molecular mass of 10 kD. In Figure 3c, these bands are not visible, possible due to water content of the sample. This is another strong indication for a successful linkage of the mPEG-Amine10K to the MCPs surfaces.

**Figure 4:**
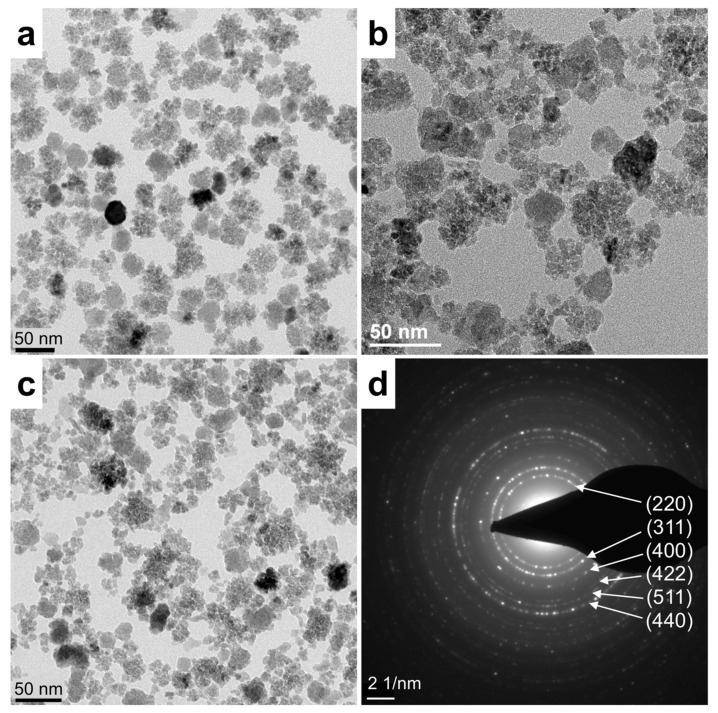
(**a**–**d**). TEM images of (**a**) MCP, (**b**) MCP-PEG10K and (**c**) MCP-PEG10K2, and (**d**) corresponding SAED pattern of MCP. Scale bar for SAED pattern is 2 nm^−1^. (For TEM image of MCP-PEG5K, please see supplement Figure S4).

Transmission electron microscopy (TEM) analysis of MCP and the two pegylated variants, MCP-PEG10K and MCP-PEG10K2, show the typical multicore structure of MCP (Figure 4), which is also preserved in the pegylated MCP [25]. It seems that there are only small differences between the parent MCPs and the pegylated MCPs in TEM, such as a possibly smaller degree of aggregation during grid preparation, and the difference is likely to be greater for the doubly pegylated variant. Note though that the difference might also be attributable to the selection of different areas for TEM. Therefore, further, more detailed investigations like x-ray diffraction (XRD), Mössbauer and small and wide-angle X-ray scattering (SAXS, WAXS) are needed, to determine the exact crystallographic phase and core structure of MCP. In particular, the latter is still unknown but seems to be fundamentally different from that of single core particles [25]. The selected area electron diffraction (SAED) pattern of MCPs (Figure 4d) indicates that the MNPs consist of magnetite/maghemite.

**Figure 5:**
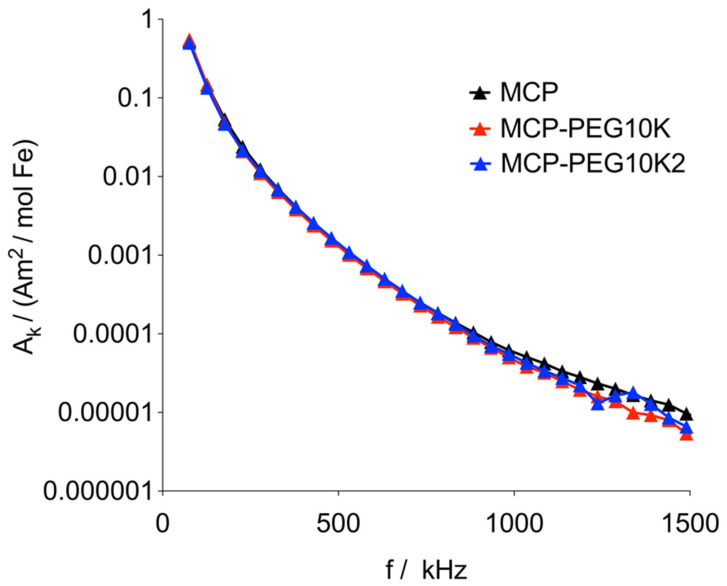
MPS results for MCP, MCP-PEG10K and MCP-PEG10K2 at 12 mT and 25 kHz. Data are plotted as magnetic moment (normalized to iron content) versus frequency. Only odd harmonics are shown, and lines have been added to guide the eye.

The MNPs were also measured by MPS, which can be regarded as a zero-dimensional MPI scanner without spatial resolution [42]. The amplitudes of the harmonics, normalized to the iron amount in the sample, of the pegylated and doubly pegylated MCPs at 12 mT and 25 kHz were almost identical, indicating that pegylation has no significant influence on the dynamic magnetic properties of the core structure (Figure 5). Conversely, for MCP-PEG5K, the amplitudes of the harmonics were significantly reduced (for MPS data of MCP-PEG5K, please see supplement Figure S5). This might have been caused by partial MNP aggregation during the synthesis process or by the presumably insufficient stabilizing effect of the 5kD peg chains. The large magnetic core diameters of MCPs need effective stabilization to prevent aggregation [25]. Once aggregated, the relatively large magnetic moments of the MCPs interact within an aggregate via a dipole–dipole interaction, that often slows down the dynamics of the moments [43]. If the subsequently increased lag of the moment behind the excitation field is large enough, the MPS amplitude decreases.

**Figure 6:**
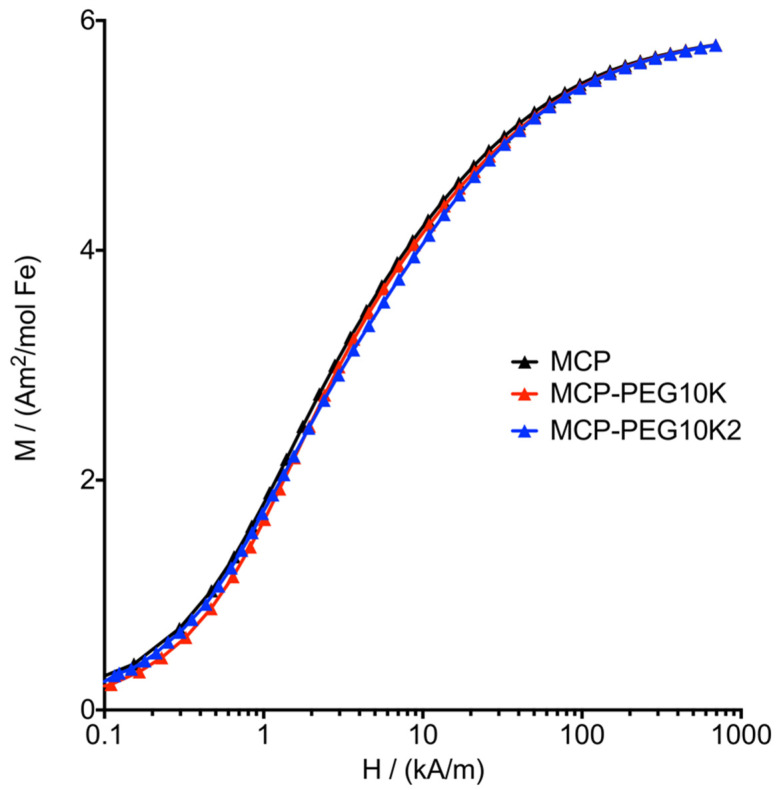
Molar magnetization *M* as a function of applied external field *H* measured for MCP, MCP-PEG10K and MCP-PEG10K2 at 295 K. For a better comparison of the curve shapes, the data of MCP-PEG10K2 were multiplied by 0.93.

The saturation magnetization (*M*_s_) of MCP-PEG10K and MCP-PEG10K2 is also as high as that of MCP, within the range of the uncertainty of 4% (sample adjustment + concentration). We assume that the atomic structure of the magnetic phase did not change during the pegylation steps. If this assumption is true, the M (H) curves of the different samples have to match within the field range of saturation. While *M*_s_ of MCP and MCP-PEG10K (Note: full saturation is not achieved at H = 790 kA/m) is 5.87 Am^2^/mol (Fe) and 5.75 Am^2^/mol (Fe), respectively, that of MCP-PEG10K2 is 6.18 Am^2^/mol (Fe). The last value could be slightly overestimated. This was concluded from the good agreement of the MPS curves (Figure 5). Thus, this difference is due to a to a slight deviation of the sample from the center position in the M (H) measurement rather than an underestimation of iron content. Accordingly, we multiplied the M (H) curve of MCP-PEG10K2 by 0.93, yielding a match of the M (H) data at high fields. This way, differences between the M(H) curves in the lower and middle field range of the curve become apparent. Here, the curves differ only slightly in shape (Figure 6). From this, we conclude that neither the magnetic cores of the MCPs nor their size distribution changed significantly after pegylation. The observed slight alterations indicate very small changes in core size distribution, probably introduced by the additional washing steps.

### 3.2. Animal Blood Half-Life Determination Using MRI

#### 3.2.1. Blood Half-Life Determination of MCP-PEG10K

**Figure 7:**
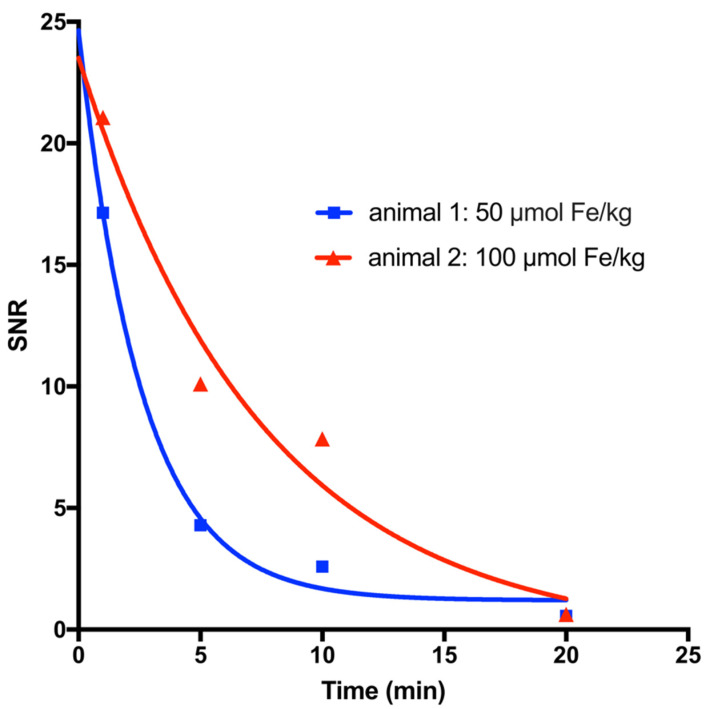
T1-weighted half-life of MCP-PEG10K measured using MRI in rats administered doses of 50 and 100 µmol Fe/kg. Scheme 1. 8 and 5.2 min, respectively.

For a first evaluation of the blood half-life of MCP-PEG10K, MNPs were administered into the tail vein of two rats at doses of 50 and 100 µmol Fe/kg. The rats were then examined in a 1.5 Tesla MRI scanner to measure transient intravascular signal enhancement over time. The blood half-life of MCP-PEG10K was 1.8 and 5.2 min at 50 and 100 µmol Fe/kg, respectively (Figure 7). The half-life was significantly reduced compared to similar MCPs without pegylation, for which 8.8 and 17.4 min at 50 and 100 µmol Fe/kg have been reported [25]. Based on these results, we synthesized MCP-PEG10K2 by a second reaction of MCP-PEG10K with mPEG-amine10K to further increase the PEG density on the MCPs surfaces.

#### 3.2.2. Blood Half-Life Determination of MCP-PEG10K2

**Figure 8:**
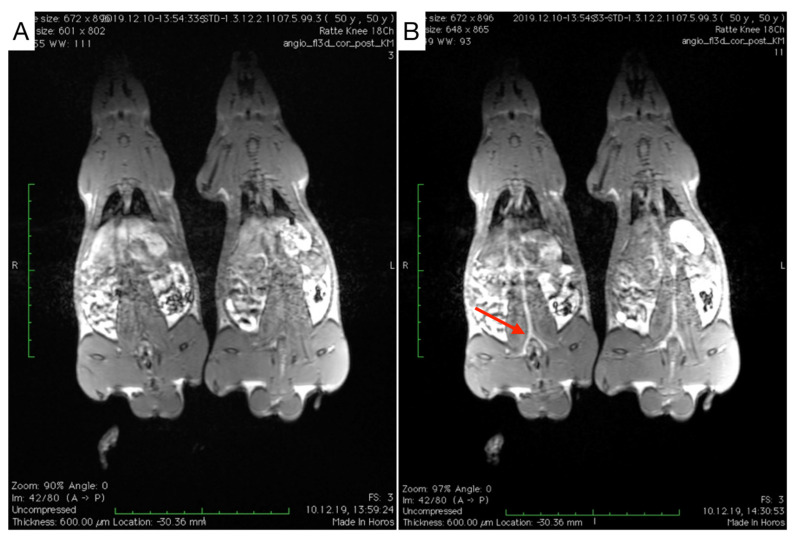
T1-weighted MR images of 2 male Sprague Dawley rats before (**A**) and 20 min after administration (**B**) of MCP-PEG10K2 (50 µmol Fe/kg). The red arrow in B indicates the area where signal enhancement was measured to determine the blood half-life of MCP-PEG10K2.

For determination of the blood half-life of MCP-PEG10K2, MNP were administered to a total of three healthy 12-week-old male Sprague Dawley rats and measured in the then newly installed 3 Tesla MRI scanner using a T1-weighted pulse sequence after administration of a dose of 50 µmol Fe/kg (Figure 8).

**Figure 9:**
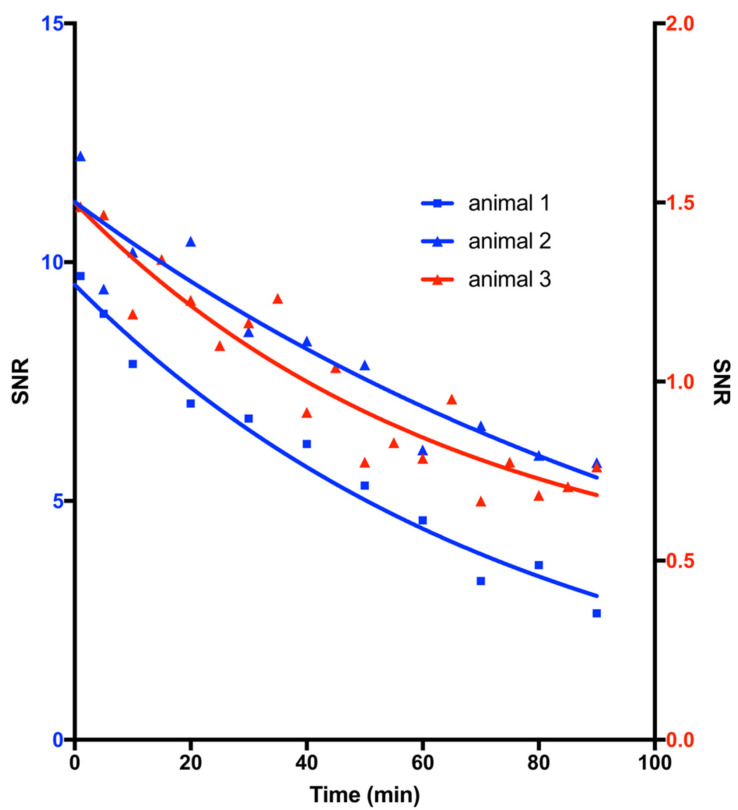
T1-weighted half-life of MCP-PEG10K2 measured using MRI in rats following administration of a dose of 50 µmol Fe/kg. Shown is the course of MRI signal intensity over time. The blood half-lives calculated from the measured data were 54.1 min for animal 1, 86.8 min for animal 2 and 45.4 min for animal 3.

The blood half-life of MCP-PEG10K2 determined by T1w MRI in three measurements was 45.4, 54.1 and 86.8 min, from which a mean blood half-life of 62.1 min was calculated (Figure 9).

To explain the totally different in vivo behavior of MCP-PEG10K and MCP-PEG10K2 one should take a closer look at what happens in vivo with MNPs in general. After intravenous injection of a tracer, serum/complement proteins are rapidly adsorbed on the surface of the MNPs [44,45]. This process is known as opsonization and the adsorbed proteins are called opsonins [46] and is driven by the tendency of the body fluid/MNP system to minimize free enthalpy, also known as Gibbs energy [45]. Consequently, in general, the rapidly formed protein shell, or protein corona, gives the MNPs their in vivo biological identity and fundamentally changes the initial surface properties, thus determining the subsequent in vivo behavior of the MNPs [44,47]. On the other hand, the initial surface properties such as surface charge and lipophilicity/hydrophilicity determine which kinds of proteins are absorbed. It is assumed that opsonins on an MNP surface mostly boost their recognition by the reticuloendothelial system (RES) and thus enhance phagocytosis [31]. This can be advantageous if, for example, MNPs are administered to track macrophages, while, for other applications such as selective targeting or cardiovascular imaging, a sufficiently long blood half-life is urgently required. One common method to extend blood half-life is to cover the MNPs surfaces with PEG, which strongly reduces the interaction of the MNPs surfaces with opsonins [31]. Many reports in the literature describe a higher effectiveness of protein shielding for high-molecular-weight PEG chains, although the resulting PEG density on the MNPs surfaces is significantly lower compared to low-molecular-weight PEG chains [35,48]. The “conformational cloud” generated by the large number of possible conformations of high-molecular-weight PEG chains and fast transition have been reported to reduce the surface charge and protein interactions [29]. The effectiveness of this stealth effect is not only dependent on the molecular weight of the PEG polymer but is also strongly influenced by its surface density. It has been shown for pegylated gold nanoparticles that there is an optimum of PEG surface density, above which protein resistance decreases [49]. The conversion of MCPs with mPEG-amine10K leads to stable dispersions of MCP-PEG10K with a surface charge near zero. Nevertheless, the MRI blood half-life of MCP-PEG10K was shorter in rats compared with unpegylated MCPs, which means that uptake by the RES was increased after the first conversion with PEG and not reduced. This may have been caused by a relatively low PEG density on the MNPs surfaces and a resulting “mushroom” or “brush” configuration of the PEGs bound to the surface. Such a configuration is not dense enough to ensure sufficient shielding of the MNPs surfaces [50]. This could lead to the accumulation of proteins on the particle surface, which promote increased uptake by the RES. The longer blood half-life of MCP-PEG10K2 measured in rats after the second conversion with mPEG-amine10K is probably attributable to a higher PEG density on the MNPs surfaces, which was also confirmed by our results of the physicochemical characterization.

### 3.3. Determination of Organ Distribution of MCP-PEG10K2 by MPS

**Figure 10:**
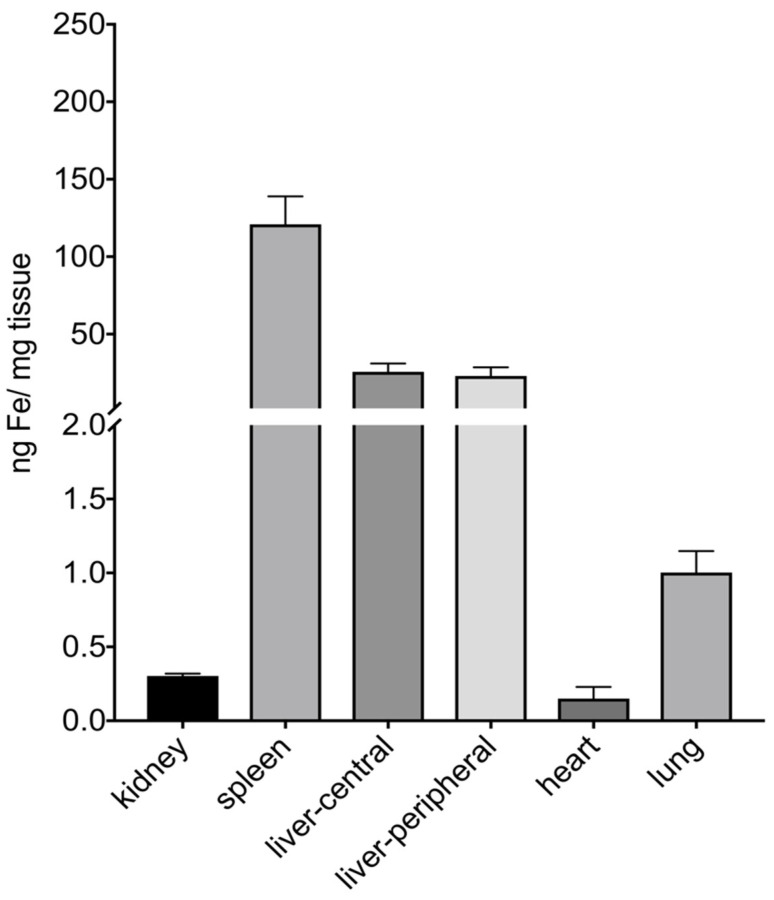
Postmortem organ distribution of MCP-PEG10K2 in three rats, 24 h after administration of 50 µmol/kg MCP-PEG10K2 determined by MPS (25 mT, 25 kHz and 37 °C). The results of measurements in organs of treated and untreated animals (control) are summarized in supplement Tables S2 and S3.

MR images of the rats acquired after 24 h were assessed for visual qualitative determination of MNP organ distribution. The results indicate that MNPs are mainly taken up in the liver and spleen [51]. Afterwards, the rats were euthanized and the organs were removed, cut into small pieces, and the MNP content was quantified by MPS. The highest concentrations of MNPs were found in the spleen and liver and only very low concentrations in the kidneys, heart and lungs (Figure 10).

The organ distribution of MCPs quantified by MPS and the qualitative results obtained from T2 *-weighted MR images of liver and spleen (for MR images, please see supplement Figure S7) are similar to those reported for pegylated MNPs synthesized by thermal decomposition [52].

## 4. Conclusions and Outlook

We successfully modified MCPs for use as a blood pool MPI tracer using a surface pegylation strategy. After a second conversion with mPEG-amine10K, the resulting doubly pegylated MCP-PEG10K2 showed a significantly extended blood half-life measured by MRI in rats, indicating that an adequate PEG surface density was accomplished. In addition, our work is also a first proof of principle that surface modification can be achieved while at the same time preserving the excellent magnetic properties of our MCPs with a view to meeting the specific requirements of new MPI applications. In the future, the same technique can now be used to modify the particle surface of MCPs with amino-group-bearing molecules such as peptides, antibodies, proteins in general, drugs or functionalized PEG chains, to name just a few [53,54]. The significantly prolonged MRI blood half-life makes MCP-PEG10K2 a very promising blood pool MPI tracer. Therefore, the next step will be to test MCP-PEG10K2 in vivo for cardiovascular MPI imaging.

## Figures and Tables

**Table 1:** MCP-PEG variants obtained by conversion of MCPs with different mPEG-amines.

MCP-PEG Variant	Dispersion Stability
MCP-PEG2K	not stable
MCP-PEG5K	low (sedimentation after 6 h)
MCP-PEG10K	stable
MCP-PEG10K2	stable
MCP-PEG20K	low (sedimentation after 2 h and detection of aggregates)

**Table 2:** Properties of the pegylated MCPs and the parent MCP. (For corresponding data of MCP-PEG5K please see supplement Table S1).

Tracer	r1 L mmol^−1^ s^−1^	r2 L mmol^−1^ s^−1^	d_V_ DLS nm	Z-Average nm	PdI	ζ-Potential [mV]
MCP	21	346	40.8	49.4	0.114	−38.9
MCP-PEG10K	19	404	64.0	78.5	0.126	−3.2
MCP-PEG10K2	22	427	84.1	103.0	0.128	−3.4

## Data Availability

The data presented in this study are available on request from the corresponding author.

## Supplementary Materials

The following files are available online at https://www.mdpi.com/article/10.3390/nano11061532/s1: Protocol S1: Synthesis of MCP-PEG2K; Protocol S2: Synthesis of MCP-PEG20K; Figure S1: DLS data (intensity) of MCP, MCP-PEG10K and MCP-PEG10K2; Figure S2: DLS data (volume) of MCP-PEG5K and MCP-PEG20K; Figure S3: DLS data of MCP-PEG5K and MCP-PEG20K (intensity); Figure S4: TEM image of MCP-PEG5K; Figure S5: MPS data of MCP and MCP-PEG5K at 12 mT and 25 kHz; Figure S6: MPS data of MCP-PEG10K2 in aqueous dispersion and polyacrylamide (PAA) gel at 25 mT and 25 kHz; Figure S7: Qualitative biodistribution of MCP-PEG10K2 in representative T2 * w MR images before and after injection (24 h) of 50 µmol Fe/kg; Table S1: Compilation of properties of MCP-PEG5K; Table S2: Post mortem MPS measurements in organs of 3 rats, 24 h after administration of 50 µmol/kg MCP-PEG10K2 (MPS parameters: 25 mT, 25 kHz and 37 °C); Table S3: Post mortem MPS measurements in organs of 3 untreated control rats (MPS parameters: 25 mT, 25 kHz and 37 °C); Table S4: Characteristic FTIR absorption bands of MCP; Table S5: Characteristic FTIR absorption bands of mPEG-Amine10K; Table S6: Characteristic FTIR absorption bands of MCP-PEG10K; Table S7: Characteristic FTIR absorption bands of MCP-PEG10K2.
