# Supplementary material for: Tailored Magnetic Multicore Nanoparticles for Use as Blood Pool MPI Tracers
Source: Nanomaterials (Basel). 2021 Jun 10;11(6):1532. doi: 10.3390/nano11061532 (PMC8228684; doi:10.3390/nano11061532)
Supplement: Supplementary file 1 [file nanomaterials-11-01532-s001.zip › nanomaterials-1209175-supplementary.pdf]

nanomaterials

Supplementary Materials

Tailored Magnetic Multicore Nanoparticles for Use as Blood Pool MPI Tracers

Harald Kratz[1]*, Azadeh Mohtashamdolatshahi[1], Dietmar Eberbeck[2], Olaf Kosch[2], Frank Wiekhorst[2], Matthias Taupitz[1], Bernd Hamm[1], Nicola Stolzenburg[1] and Jörg Schnorr[1].

[1] Department of Radiology, Charité-Universitätsmedizin Berlin, corporate member of Freie Universität Berlin and Humboldt-Universität zu Berlin, D-10117 Berlin, Germany; Azadeh.Mohtashamdolatshahi@charite.de (A.M.); Matthias.Taupitz@charite.de (M.T.); Bernd.Hamm@charite.de (B.H.); Nicola.Stolzenburg@charite.de (N.S.); Joerg.Schnorr@charite.de (J.S.)

[2] Physikalisch-Technische Bundesanstalt, D-10587 Berlin, Germany; Dietmar.Eberbeck@ptb.de (D.E.); Olaf.Kosch@ptb.de (O.K.); Frank.Wiekhorst@ptb.de (F.W.)

* Correspondence: Harald.Kratz@charite.de; Tel.: +49-30-450-527180

Protocol S1. Synthesis of MCP-PEG2K

An amount of 80 µL MCP dispersion (275 mM Fe/L, 22µmol Fe) was subsequently mixed with a solution of 20 mg (92.1 µmol) *N*-hydroxysulfosuccinimide sodium salt (sulfo-NHS) in 50 µL water and a solution of 60 mg (0.313 mmol) *N*-(3-dimethylaminopropyl)-*N*′-ethylcarbodiimide hydrochloride (EDC*HCl) in 150 µL water. Thereafter the mixture was agitated for 30 min at room temperature in a rotary blender. The particle dispersion was then concentrated using two centrifuge filters (100kD, RC, Ultracel, Amicon Ultra 0.5) at 2900 × g to a volume of 180 µL (2 x 90 µL). The concentrated dispersions were then washed four times by adding of 400 µL and following centrifugation. The washed dispersions were combined and added to a solution of 40 mg (20 µmol) mPEG-amine2K (2kD) in 200 µL of water. After thorough mixing, the resulting batch was sonicated for 20 min using a Sonorex Digital 10 P Sonicator (Bandelin, Berlin, Germany) at 100% intensity and then mixed with a Roto-Therm Plus rotary blender (Benchmark, Sayreville, NJ, USA) at room temperature overnight. The dispersions were concentrated to a volume of about 50 µl in each case using two centrifuge filters (100kD Ultracel Amicon Ulta 0,5) at 2900 x g. Then 400 µL of water was added to each filter and the dispersion again concentrated at 2900 x g to about 50 µl in each case. The last step, addition of water and centrifugation, was repeated three more times. The MNP were redispersed by rinsing the filter with water. The resulting dispersions were collected and increased to a total volume of 500 µL by the addition of water and sonicated for 20 min using a Sonorex Digital 10 P Sonicator (Bandelin, Berlin, Germany) at 100% intensity. The final dispersion was unstable.

Protocol S2. Synthesis of MCP-PEG20K

An amount of 200 µL MCP dispersion (110.8 mM Fe/L, 22µmol Fe) was subsequently mixed with a solution of 20 mg (92.1 µmol) *N*-hydroxysulfosuccinimide sodium salt (sulfo-NHS) in 50 µL water and a solution of 60 mg (0.313 mmol) *N*-(3-dimethylaminopropyl)-*N*′-ethylcarbodiimide hydrochloride (EDC*HCl) in 150 µL water. Thereafter the mixture was agitated for 30 min at room temperature in a rotary blender. The particle dispersion was then centrifuged at 900 x g for 5 sec and 400 µL of the supernatant was removed and replaced by 400 µL of water, and the mixture was vortexed. The particle dispersion was then centrifuged a second time at 900 x g for 2 sec and 400 µL of the supernatant was removed, 400 µL of water was added, and the mixture was vortexed again. After that the dispersion was added to a solution of 400 mg (20 µmol) mPEG-amine20K (20kD) in 700 µL of water, which was tempered for 30 min at 55°C. After thorough mixing, the resulting batch was sonicated for 99 min using a Sonorex Digital 10 P Sonicator (Bandelin, Berlin,

Germany) at 100% intensity (the temperature rose from room temperature to 55°C) and then mixed with a Roto-Therm Plus rotary blender (Benchmark, Sayreville, NJ, USA) at room temperature overnight. Water was then added to reach a total volume of about 30 mL, and the dispersion was concentrated to a volume of about 250 µl in each case using two centrifuge filters (100kD Ultracel Amicon Ulta 15,) at 3372 x g. Then 2 mL of water was added to each filter, and the dispersion again concentrated at 3372 x g to about 200 µl in each case. The last step, addition of water and centrifugation, was repeated three more times, and the volume of the resulting dispersion was collected and increased to a total volume of 500 µL by addition of water. Iron content for MCP-PEG20K: 32 mM Fe/L (yield: 72% related to Fe).

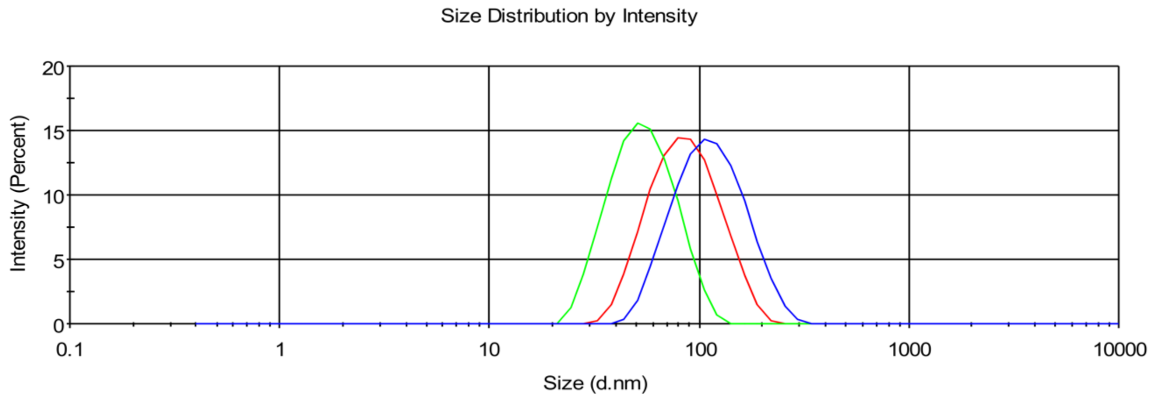

Figure S1. DLS data of MCP (green), MCP-PEG10K (red) and MCP-PEG10K2 (blue). Intensity data, mean of 6 measurements.

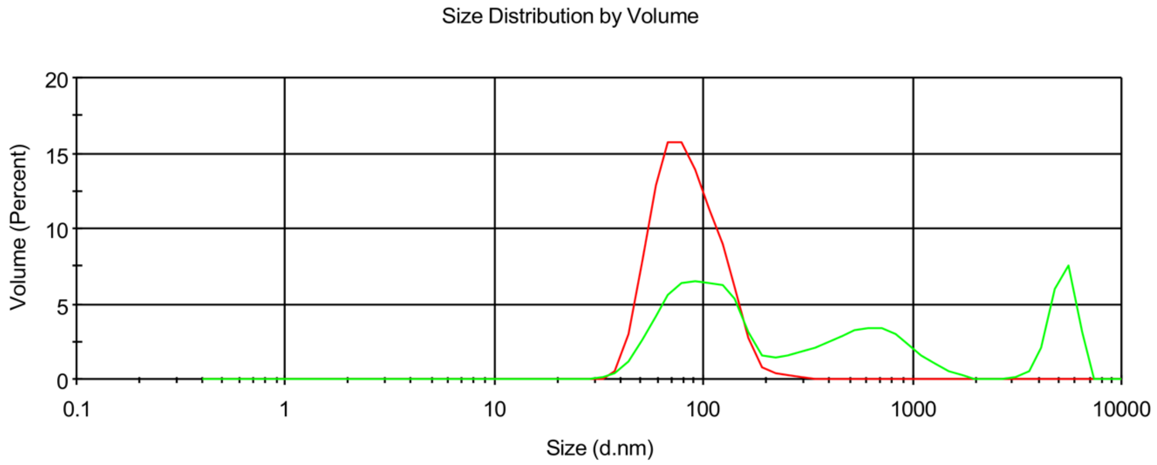

Figure S2. DLS data of MCP-PEG5K (red) and MCP-PEG20K (green). Volume data, mean of 6 measurements.

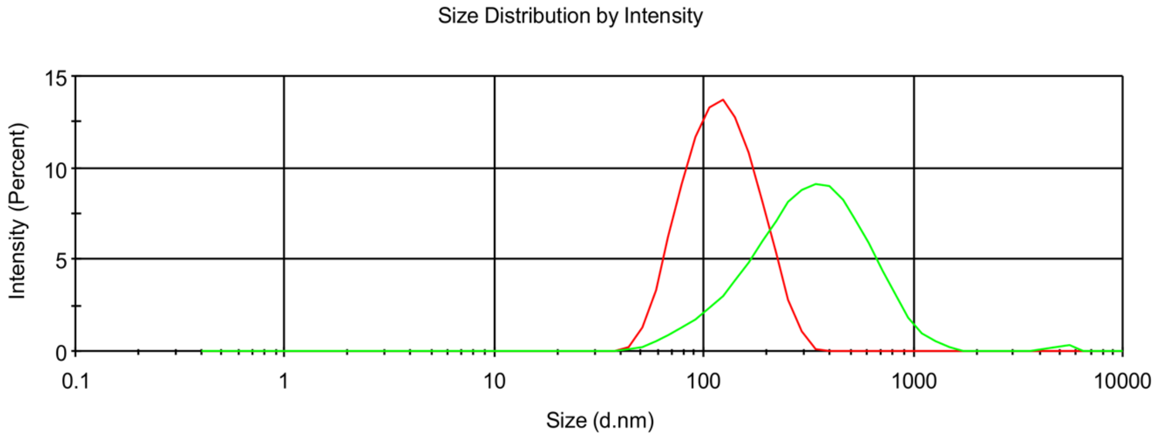

Figure S3. DLS data of MCP-PEG5K (red) and MCP-PEG20K (green). Intensity data, mean of 6 measurements.

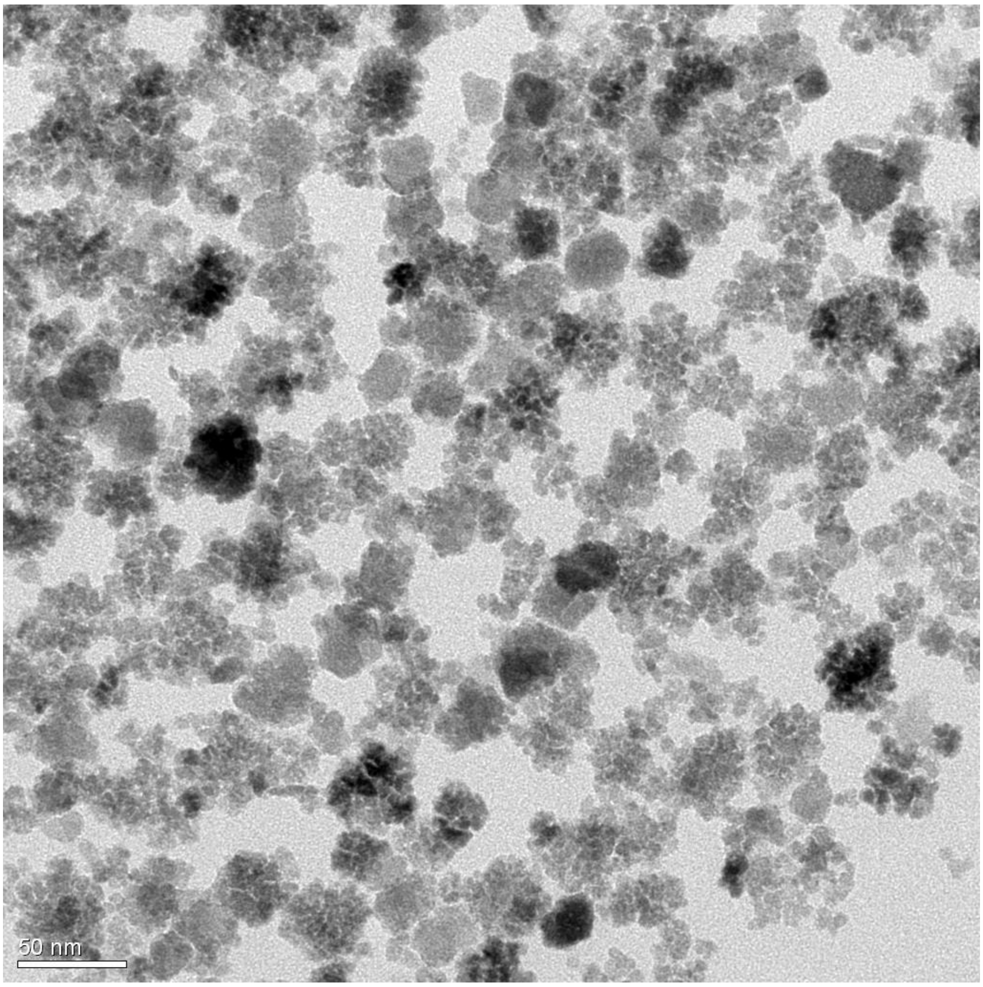

Figure S4. TEM image of MCP-PEG5K.

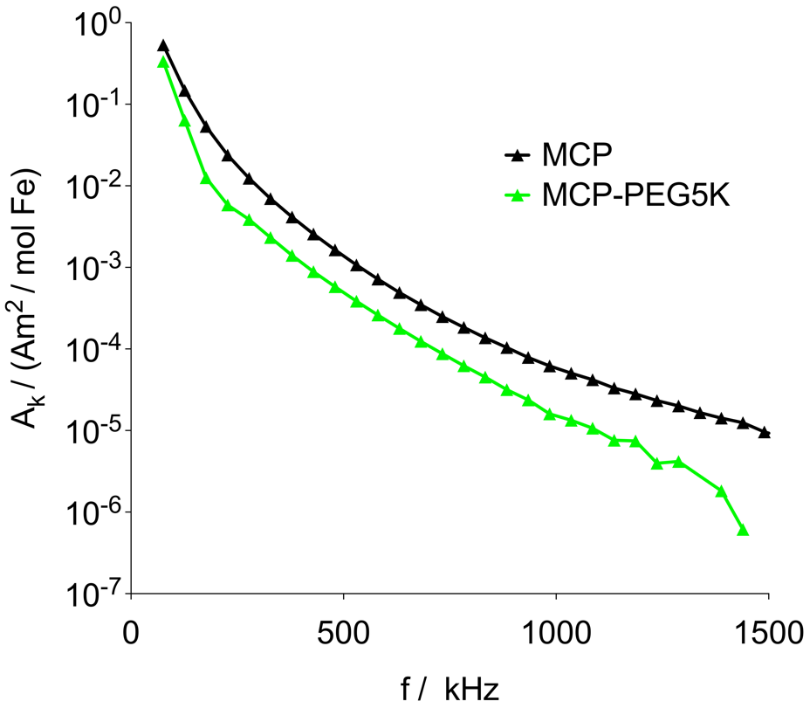

Figure S5. MPS data of MCP and MCP-PEG5K at 12 mT and 25 kHz. Data are plotted as magnetic moment (normalized to iron content) versus frequency. Only odd harmonics are shown, and lines have been added to guide the eye.

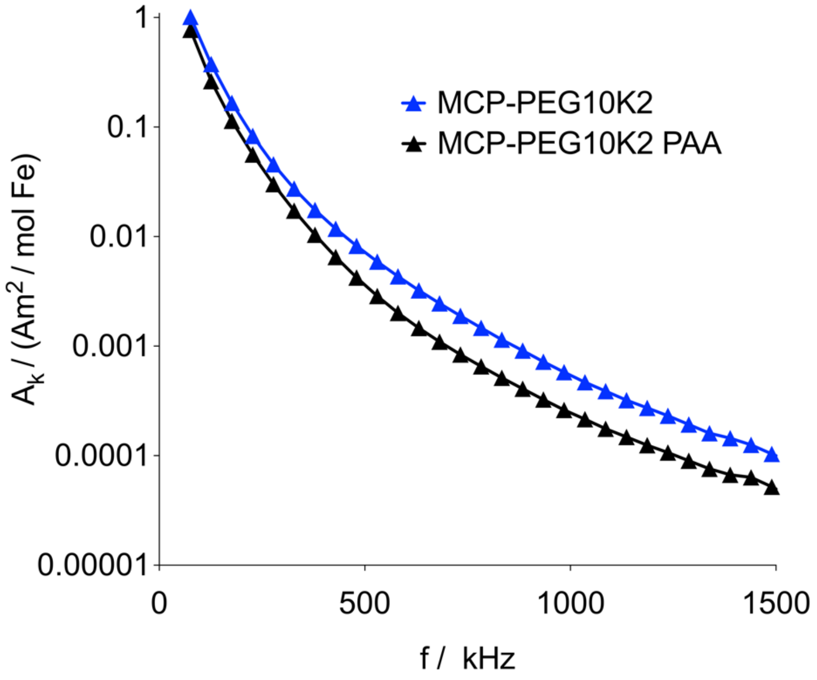

Figure S6. MPS data of MCP-PEG10K2 in aqueous dispersion (blue) and polyacrylamide (PAA) gel (black) at 25 mT and 25 kHz. The MCP-PEG10K2 PAA gel was used as a reference for organ iron quantification/ distribution. Data are plotted as magnetic moment (normalized to iron content) versus frequency. Only odd harmonics are shown, and lines have been added to guide the eye.

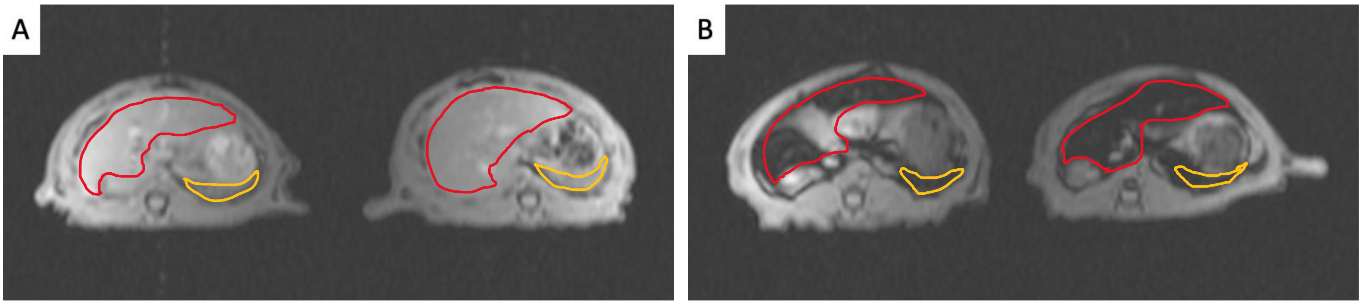

Figure S7. Qualitative biodistribution of MCP-PEG10K2 in representative T2*w MR images before (**A**) and after (**B**) injections (24h) of 50 µmol Fe/kg. Decreased signal in liver (encircled in red) and spleen (encircled in orange) indicates uptake of MCP-PEG10K2 in these two organs.

Table S1. Properties of MCP-PEG5K.

Tracer	r1 [L mmol^{-1} s^{-1}]	r2 [L mmol^{-1} s^{-1}]	dv DLS [nm]	Z-Average [nm]	Pdi	ζ-Potential [mV]
MCP-PEG5K	17	435	87.3	111.4	0.145	-9.5

Given are the longitudinal and transverse relaxation rates (r1) and (r2) measured by TD-NMR. Furthermore, the mean hydrodynamic diameter by volume (dv), the intensity-weighted mean hydrodynamic size (Z-average), the polydispersity index (PDI), and the zeta potential (ζ), all measured by DLS, are shown.

Table S2. Post mortem MPS measurements in organs of 3 rats, 24 h after administration of 50 µmol/kg MCP-PEG10K2 (MPS parameters: 25 mT, 25 kHz and 37°C). Values are given in ng Fe/mg tissue.

**organ**	**rat 1**	**rat 2**	**rat3**	**mean**
kidney	0.32	0.29	0.30	0.30
spleen	117.41	140.47	104.85	120.91
liver - central	26.35	30.69	20.02	25.69
liver - peripheral	29.41	19.01	20.39	22.94
heart	0.24	0.09	0.12	0.15
lung	1.15	1.00	0.86	1.00

Table S3. Post mortem MPS measurements in organs of 3 untreated control rats (MPS parameters: 25 mT, 25 kHz and 37°C). Values are given in ng Fe/mg tissue.

**organ**	**rat 1**	**rat 2**	**rat3**	**mean**
kidney	0.03	0.01	0.00	0.013
spleen	0.02	0.02	0.01	0.017
liver - central	0.00	0.01	0.00	0.003
liver - peripheral	0.00	0.00	0.00	0.000
heart	0.01	0.00	0.02	0.010
lung	0.04	0.13	0.02	0.063

Table S4. Characteristic FTIR absorption bands of MCP.

542 cm^{-1}: Fe-O stretch (Magnetite/ Maghemite)
1009 cm^{-1}: alkoxy C-O (CMD)
1584 cm^{-1}: carboxylate, C=O stretch and alcohol O-H, (CMD) and presumably physisorbed water.
2884 cm^{-1}: C-H stretch (CMD)
3137 cm^{-1}: alcohol O-H stretch, acid O-H stretch (CMD) and presumably physisorbed water.

Table S5. Characteristic FTIR bands of mPEG-amine10K.

841 cm^{-1}: C-H rock
953 cm^{-1}: C-H rock, twist
1062 cm^{-1}: C-C and C-O stretch, C-H rock
1096 cm^{-1}: C-C and C-O stretch
1145 cm^{-1}: C-H rock and C-O stretch
1240 cm^{-1}: C-H twist
1279 cm^{-1}: C-H twist
1341 cm^{-1}: C-H bend
1463 cm^{-1}: C-H bend
2881 cm^{-1}: C-H stretch

Table S6. Characteristic FTIR bands of MCP-PEG10K.

541 cm^{-1}: Fe-O stretch (Magnetite/ Maghemite)
843 cm^{-1}: C-H rock (PEG)
953 cm^{-1}: C-H rock, twist (PEG)
1099 cm^{-1}: C-C and C-O stretch (PEG)
1242 cm^{-1}: C-H twist (PEG)
1278 cm^{-1}: C-H twist (PEG)
1342 cm^{-1}: C-H bend (PEG)
1459 cm^{-1}: C-H bend (PEG)
1596 cm^{-1}: carboxylate, C=O stretch and alcohol O-H (CMD) and presumably physisorbed water.
2857 cm^{-1}: C-H stretch (PEG)
3165 cm^{-1}: alcohol O-H stretch, acid O-H stretch (CMD) and presumably physisorbed water.

Table S7. Characteristic FTIR bands of MCP-PEG10K2.

574 cm^{-1}: Fe-O stretch, (Magnetite/ Maghemite)
840 cm^{-1}: C-H rock (PEG)
954 cm^{-1}: C-H rock, twist (PEG)
1094 cm^{-1}: C-C and C-O stretch (PEG)
1239 cm^{-1}: C-H twist (PEG)
1279 cm^{-1}: C-H twist (PEG)
1341 cm^{-1}: C-H bend (PEG)
1464 cm^{-1}: C-H bend (PEG)
1565 cm^{-1}: N-H bend and C-N stretch (Amide II)
1649 cm^{-1}: C=O stretch (Amide I)
2880 cm^{-1}: C-H stretch (PEG)
3344 cm^{-1}: alcohol O-H stretch, acid
